# Bibliometric and visualization analysis of matrix metalloproteinases in ischemic stroke from 1992 to 2022

**DOI:** 10.3389/fnins.2023.1206793

**Published:** 2023-07-06

**Authors:** YiKun Gao, Yina Li, Shi Feng, Lijuan Gu

**Affiliations:** Renmin Hospital of Wuhan University, Wuhan, Hubei, China

**Keywords:** ischemic stroke, matrix metalloproteinases, bibliometric, Citespace, VOSviewer

## Abstract

**Background:**

Matrix metalloproteinases (MMPs) are important players in the complex pathophysiology of ischemic stroke (IS). Recent studies have shown that tremendous progress has been made in the research of MMPs in IS. However, a comprehensive bibliometric analysis is lacking in this research field. This study aimed to introduce the research status as well as hotspots and explore the field of MMPs in IS from a bibliometric perspective.

**Methods:**

This study collected 1,441 records related to MMPs in IS from 1979 to 2022 in the web of science core collection (WoSCC) database, among them the first paper was published in 1992. CiteSpace, VOSviewer, and R package “bibliometrix” software were used to analyze the publication type, author, institution, country, keywords, and other relevant data in detail, and made descriptive statistics to provide new ideas for future clinical and scientific research.

**Results:**

The change in the number of publications related to MMPs in IS can be divided into three stages: the first stage was from 1992 to 2012, when the number of publications increased steadily; the second stage was from 2013 to 2017, when the number of publications was relatively stable; the third stage was from 2018 to 2022, when the number of publications began to decline. The United States and China, contributing more than 64% of publications, were the main drivers for research in this field. Universities in the United States were the most active institutions and contributed the most publications. STROKE is the most popular journal in this field with the largest publications as well as the most co-cited journal. Rosenberg GA was the most prolific writer and has the most citations. “Clinical,” “Medical,” “Neurology,” “Immunology” and “Biochemistry molecular biology” were the main research areas of MMPs in IS. “Molecular regulation,” “Metalloproteinase-9 concentration,” “Clinical translation” and “Cerebral ischemia–reperfusion” are the primary keywords clusters in this field.

**Conclusion:**

This is the first bibliometric study that comprehensively mapped out the knowledge structure and development trends in the research field of MMPs in IS in recent 30 years, which will provide a reference for scholars studying this field.

## 1. Introduction

Stroke is the main clinical type of cerebrovascular disease, including ischemic stroke (IS) and hemorrhagic stroke (HS). It is a group of cerebrovascular diseases caused by organic brain injury and brain cell death, with the common clinical characteristics of sudden onset and rapid localized or diffuse brain function defect. Stroke is currently the second leading cause of human death and the leading disabling disease in adults, and IS accounts for the majority (Writing Group et al., 2016). IS occurs when blood flow to a part of the brain is temporarily or permanently restricted, which causes irreversible damage to the ischemic core and surrounding areas of potentially recoverable tissue. Matrix metalloproteinases (MMPs) are zinc-dependent endopeptidases in the polyene family of more than 25 enzymes, most of which are secreted as catalytic latent substances and act extracellular (Beroun et al., 2019). They are important players in the complex pathophysiology of IS, including atherosclerotic plaque maturation, plaque degradation and rupture, brain edema and hemorrhagic transformation due to the opening of the blood–brain barrier (BBB), and subclinical periventricular white matter disease (Nikkari et al., 1995; Lenglet et al., 2013; Chaturvedi and Kaczmarek, 2014). In summary, MMPs play an important role in the pathological process of IS, its expression is associated with IS injury and recovery.

Bibliometrics is a cross-disciplinary discipline that uses statistical and mathematical methods to quantify all knowledge carriers and understand the foundation and frontier of the research field (van Eck and Waltman, 2017). With VOSviewer, CiteSpace, and R package “bibliometrix,” the systematic and metrological characteristics of the literature can be analyzed and studied to provide evidence for the impact of the research field. Bibliometrics has been widely used in the medical field in recent years, and many scholars have also applied it to the areas related to stroke. Such as bibliometric analysis of ferroptosis (Chen et al., 2021), inflammasomes (Yin et al., 2021) in stroke, and autophagy in IS (Song et al., 2022).

But to the best of our knowledge, there has been no bibliometric study of MMPs in IS so far. Given the important role of MMPs in the pathological process of IS, it is necessary to analyze and elucidate the current development status and prospects of this field from the perspective of bibliometrics. Through bibliometric methods, the research trends, major research institutions, research hotspots, and frontier directions in this field can be comprehensively analyzed and evaluated. This will help to better understand the current research status and future development direction in this field, and provide new ideas and directions for relevant scientific research. This paper searched the literature related MMPs in IS, analyzed the publication type, author, institution, country, keywords, and other relevant data in detail, and made descriptive statistics to provide new ideas for future clinical and scientific research.

## 2. Materials and methods

### 2.1. Data source and search strategy

**Figure 1:**
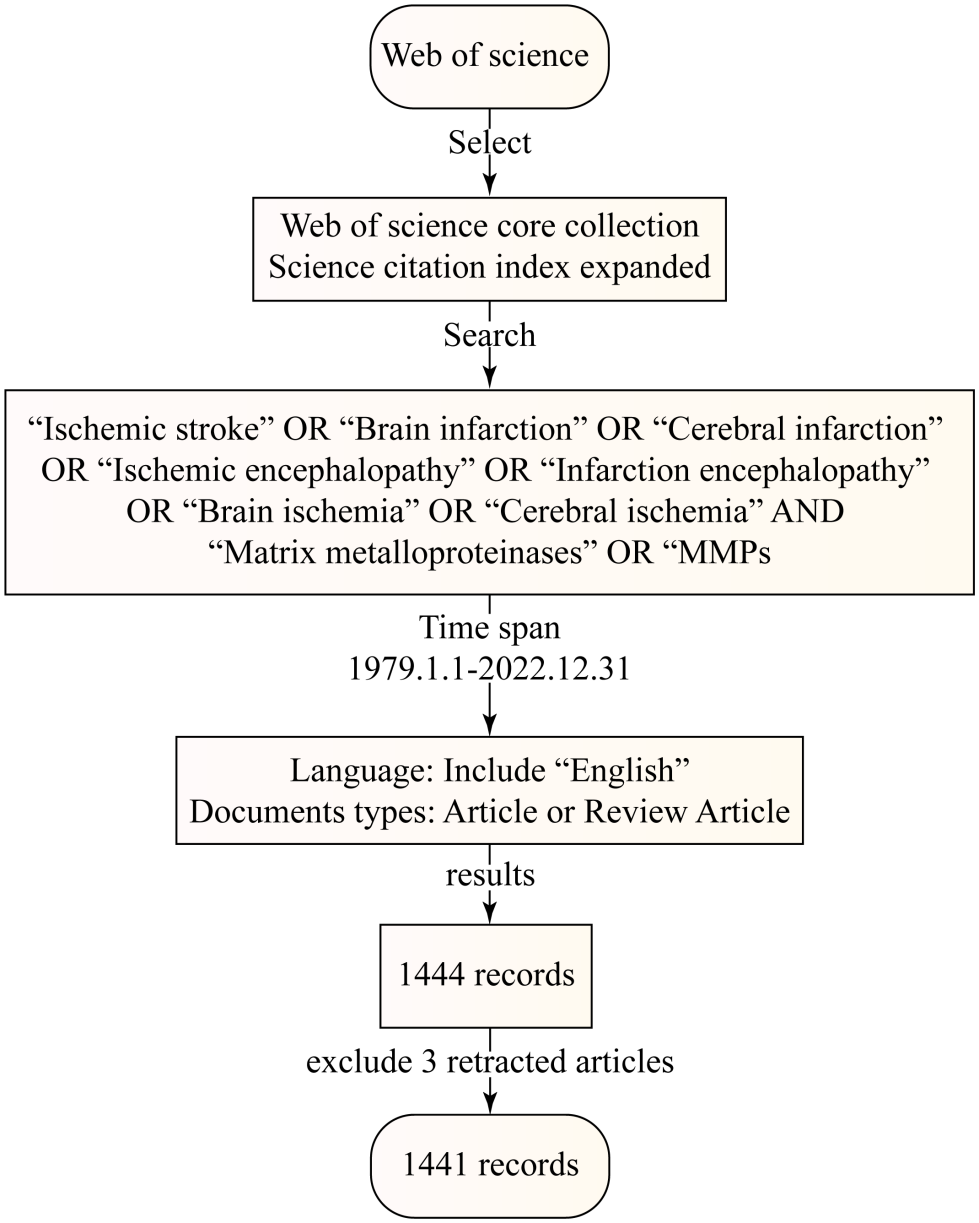
Search flowchart detailed steps in the identification and screening of papers.

Web of science core collection (WoSCC) database originating from Clarivate Analytics was considered one of the most authoritative and comprehensive database platforms. Therefore, we selected it to obtain global academic information for bibliometric analysis according to previous studies. All the published literature was extracted from WOS Science Citation Index Expanded (SCI-EXPANDED) and the date of the search was from 1 January 1979 to 31 December 2022. To produce as many results as possible, we used the following search terms: TS = [(ischemic stroke OR brain infarction OR cerebral infarction OR ischemic encephalopathy OR infarction encephalopathy OR brain ischemia OR cerebral ischemia) AND (matrix metalloproteinases OR MMPs)]. The language was restricted to English and the document types were article or review article. Following the above criteria, we obtained 1,444 original records, and the first paper was published in 1992. After excluding the 3 retracted articles, we finally obtained 1,441 results. The flow chart of the whole process is shown in Figure 1.

### 2.2. Bibliometric analysis and visualization

We use GraphPad Prism 9 for data plotting and statistical analysis. And we chose the VOSviewer (1.6.18) software to construct and visualize bibliometric networks of the publications in our present study. And the VOSviewer was performed for analyzing the bibliographic coupling, co-citation, and co-occurrence analyses in detail. In addition, we choose the R package “bibliometrix” (4.1.1) to visualize the collaboration network of highly prolific authors, visualize publications production among countries, map the international collaboration between countries, and visualize a three-field plot analysis. Moreover, CiteSpace (6.1. R6) which was developed by Professor Chen C, was used to construct a dual-map overlay for journals, cluster analysis of co-cited keywords, and detection of keywords with intense citation bursts.

## 3. Results

### 3.1. Analysis of publication types and quantities

**Figure 2:**
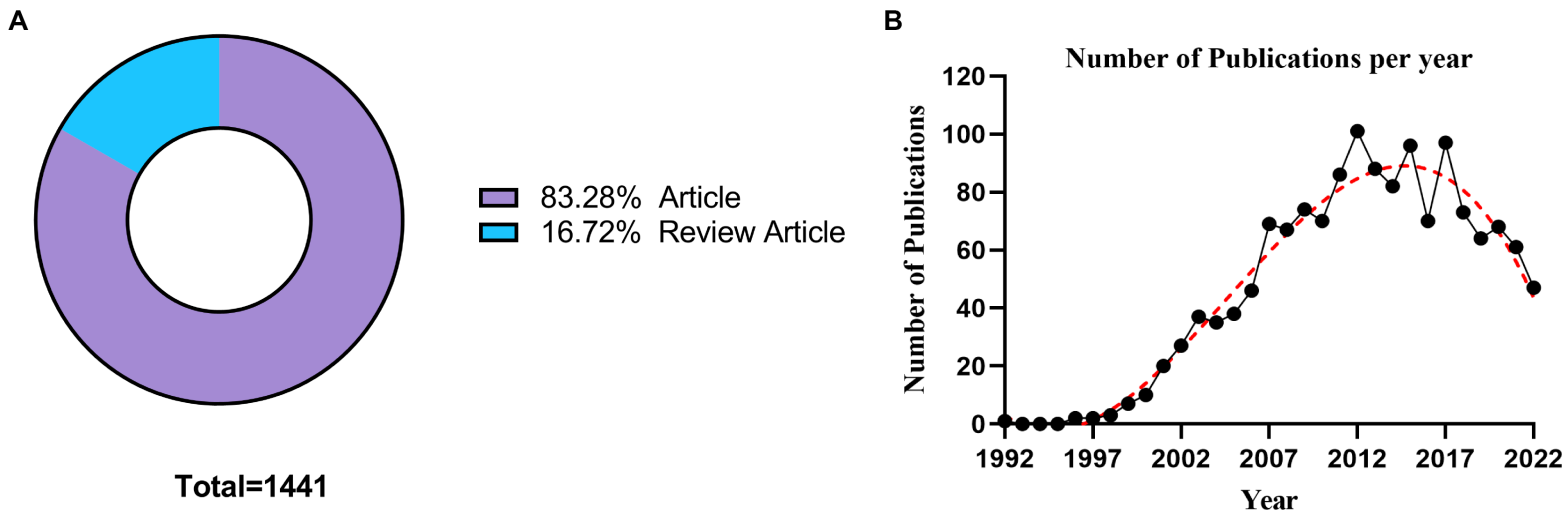
**(A)** The hollow pie chart illustrates the proportion of document numbers of each type. **(B)** The line graph shows the number of papers published on MMPs and IS studies and the polynomial trend line over time (black and red dashed lines).

A total of 1,441 publications related to the role of MMPs in IS were identified based on the search criteria, including Article (1200) and Review Article (241), these data were visualized using GraphPad Prism 9, and the results are shown in Figure 2A. The temporal distribution of published publications was visualized using GraphPad Prism 9, and the results are shown in Figure 2B. It can be seen that the number of publications first increased and then decreased. The number of publications increased rapidly from 1998 (3, 0.21%) to 2012 (101, 7.01%), however, the trend of the number of publications leveled off from 2012 to 2017 (97, 6.73%) and even showed a decrease trend from 2017 to 2022 (47, 3.26%). This suggests that the role of MMPs in IS continued to increase in heat between 1998 and 2012 and was once a hotter topic, but its heat has gradually receded in the last 5 years. In addition, Figure 2B illustrates the fitted curve for the annual publication trend, and the correction coefficient R^2^ is 0.9610. It is expected that studies related to the role of MMPs in IS will continue to be less attractive to researchers in the future.

### 3.2. Analysis of the highest cited articles

**Figure 3:**
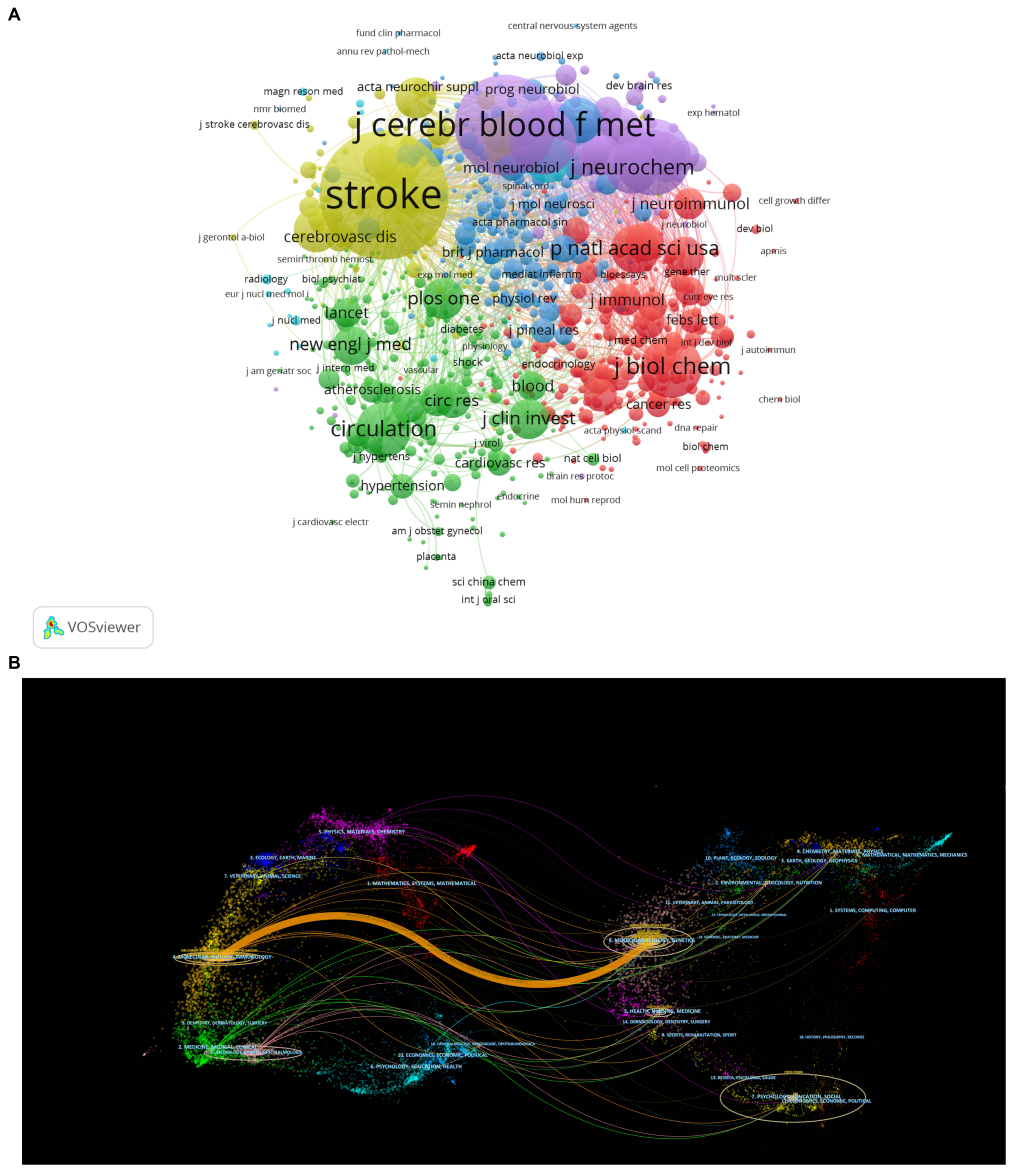
**(A)** Network map of journals that were co-cited in more than 10 citations. **(B)** The dual-map overlay of journals related to the role of MMPs in IS.

**Figure 4:**
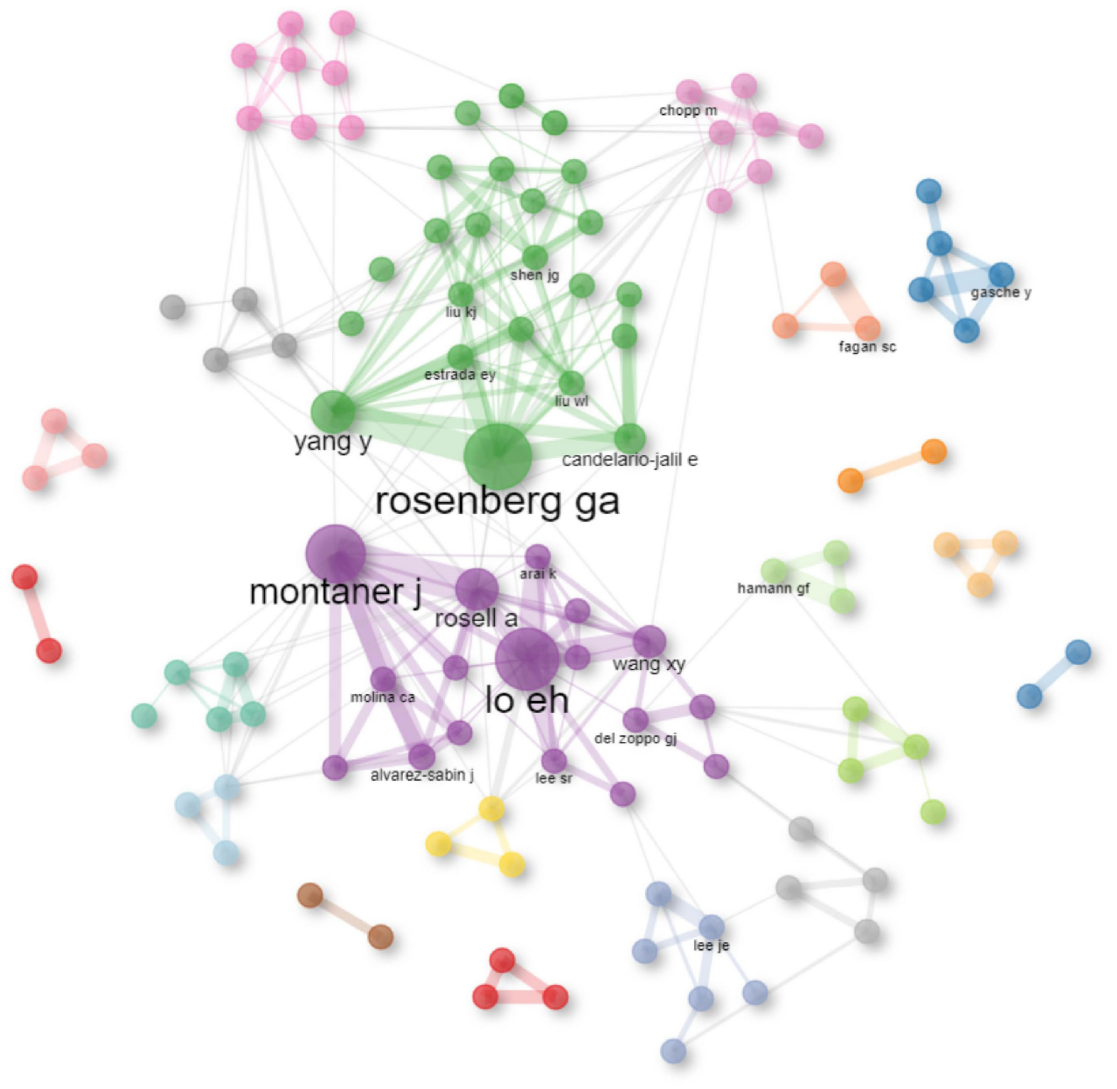
Collaboration Network of Highly Prolific Authors with ≥6 publications. The larger the node, the higher the posting volume; the thicker the line, the closer the cooperation. The color represents clustering, and nodes with the same color belong to the same.

The local citations of a document indicate the number of times the document has been cited by other documents under the same topic. An analysis of the top 10 reviews and articles with the most local citations under the topic “the role of MMPs in IS” was conducted. For the 10 reviews, their local citations ranged from 53 to 167, as shown in Table 1A. For the 10 articles, their local citations ranged from 133 to 299, as shown in Table 1B. Due to their high number of local citations, they can be said to be classical literature in the field and have considerable reference value (Figures 3, 4).

**Table 1A:** The Top 10 highly local cited reviews.

Rank	Title	Local citations	Authors	Source	Year
1	Matrix metalloproteinases in neuroinflammation	167	Rosenberg GA	Glia	2002
2	Metalloproteinases in biology and pathology of the nervous system	136	Yong VW	Nat Rev Neurosci	2001
3	Multiple roles for MMPs and TIMPs in cerebral ischemia	126	Cunningham LA	Glia	2005
4	Matrix metalloproteinases in cerebrovascular disease	120	Mun-Bryce S	J Cerebr Blood F Met	1998
5	Diverse roles of matrix metalloproteinases and tissue inhibitors of metalloproteinases in neuroinflammation and cerebral ischemia	119	Candelario-Jalil E	Neuroscience	2009
6	Extracellular proteolysis in brain injury and inflammation: role for plasminogen activators and matrix metalloproteinases	85	Lo EH	J Neurosci Res	2002
7	Multiphasic roles for matrix metalloproteinases after stroke	74	Rosell A	Curr Opin Pharmacol	2008
8	Matrix metalloproteinases and blood–brain barrier disruption in acute ischemic stroke	57	Lakhan SE	Front Neurol	2013
9	Blood–brain barrier breakdown in acute and chronic cerebrovascular disease	55	Yang Y	Stroke	2011
10	Matrix metalloproteinase-9 as a marker for acute ischemic stroke: a systematic review	53	Ramos-Fernandez M	J Stroke Cerebrovasc	2011

**Table 1B:** The top 10 highly local cited articles.

Rank	Title	Local citations	Authors	Source	Year
1	Matrix Metalloproteinases and TIMPs Are Associated With Blood–Brain Barrier Opening After Reperfusion in Rat Brain	299	Rosenberg GA	Stroke	1998
2	Matrix Metalloproteinase Expression Increases After Cerebral Focal Ischemia in Rats	261	Romanic AM	Stroke	1998
3	Role for Matrix Metalloproteinase 9 after Focal Cerebral Ischemia: Effects of Gene Knockout and Enzyme Inhibition with BB-94	231	Asahi M	J Cerebr Blood F Met	2000
4	Matrix Metalloproteinase-Mediated Disruption of Tight Junction Proteins in Cerebral Vessels is Reversed by Synthetic Matrix Metalloproteinase Inhibitor in Focal Ischemia in Rat	205	Yang Y	J Cerebr Blood F Met	2007
5	Matrix Metalloproteinases Increase Very Early during Experimental Focal Cerebral Ischemia	201	Heo JH	J Cerebr Blood F Met	1999
6	Role of matrix metalloproteinases in delayed cortical responses after stroke	193	Zhao BQ	Nat Med	2006
7	Early Appearance of Activated Matrix Metalloproteinase-9 after Focal Cerebral Ischemia in Mice: A Possible Role in Blood–Brain Barrier Dysfunction	180	Gasche Y	J Cerebr Blood F Met	1999
8	Proteolytic Cascade Enzymes Increase in Focal Cerebral Ischemia in Rat	156	Rosenberg GA	J Cerebr Blood F Met	1996
9	Immunohistochemistry of matrix metalloproteinases in reperfusion injury to rat brain: activation of MMP-9 linked to stromelysin-1 and microglia in cell cultures	138	Rosenberg GA	Brain Res	2001
10	S-nitrosylation of matrix metalloproteinases: signaling pathway to neuronal cell death	133	Gu ZZ	Science	2002

An analysis of the top 10 reviews and articles with the most global citations was conducted. For the 10 reviews, their global citations ranged from 505 to 1,341, as shown in Table 2A. For the 10 articles, their global citations ranged from 379 to 787, as shown in Table 2B. Notably, among these 20 publications, except for “The blood–brain barrier” by DANEMAN R et al. in 2015, all of them were published between 1998 and 2012, illustrating the high interest of scholars in studying the role of MMPs in IS during this period. Additionally, most of these publications focus on the blood–brain barrier and neuroinflammation, reflecting the significant impact of MMPs on the development of IS in these areas. These widely cited articles also provide valuable guidance for future research directions.

**Table 2A:** The top 10 highly global cited reviews.

Rank	Title	Global citations	Authors	Source	Year
1	The science of stroke: mechanisms in search of treatments	1,341	Moskowitz MA	Neuron	2010
2	The blood–brain barrier	1,107	Daneman R	CSH Perspect Biol	2015
3	Metalloproteinases in biology and pathology of the nervous system	846	Yong VW	Nat Rev Neurosci	2001
4	Matrix metalloproteinases in neuroinflammation	680	Rosenberg GA	Glia	2002
5	Matrix metalloproteinase inhibitors as therapy for inflammatory and vascular diseases	617	Hu JL	Nat Rev Drug Discov	2007
6	PARP-1 cleavage fragments: signatures of cell-death proteases in neurodegeneration	556	Chaitanya GV	Cell Commun Signal	2010
7	Matrix metalloproteinases (MMPs) in health and disease: an overview	542	Malemud CJ	Front Biosci-Landmrk	2006
8	Is there new hope for therapeutic matrix metalloproteinase inhibition?	513	Vandenbroucke RE	Nat Rev Drug Discov	2014
9	Blood–brain barrier breakdown in acute and chronic cerebrovascular disease	507	Yang Y	Stroke	2011
10	Inflammation after intracerebral hemorrhage	505	Wang J	J Cerebr Blood F Met	2007

**Table 2B:** The top 10 highly global cited articles.

Rank	Title	Global citations	Authors	Source	Year
1	S-nitrosylation of matrix metalloproteinases: signaling pathway to neuronal cell death	787	Gu ZZ	Science	2002
2	Matrix metalloproteinase-mediated disruption of tight junction proteins in cerebral vessels is reversed by synthetic matrix metalloproteinase inhibitor in focal ischemia in rat	770	Yang Y	J Cerebr Blood F Met	2007
3	Matrix Metalloproteinases and TIMPs Are Associated With Blood–Brain Barrier Opening After Reperfusion in Rat Brain	684	Rosenberg GA	Stroke	1998
4	Matrix Metalloproteinase Expression Increases After Cerebral Focal Ischemia in Rats	622	Romanic AM	Stroke	1998
5	Role of matrix metalloproteinases in delayed cortical responses after stroke	552	Zhao BQ	Nat Med	2006
6	Role for matrix metalloproteinase 9 after focal cerebral ischemia: effects of gene knockout and enzyme inhibition with BB-94	500	Asahi M	J Cerebr Blood F Met	2000
7	Toll-like receptor 4 is involved in brain damage and inflammation after experimental stroke	449	Caso JR	Circulation	2007
8	Matrix metalloproteinases increase very early during experimental focal cerebral ischemia	425	Heo JH	J Cerebr Blood F Met	1999
9	Matrix metalloproteinase-9 pretreatment level predicts intracranial hemorrhagic complications after thrombolysis in human stroke	412	Montaner J	Circulation	2003
10	MMP-9-positive neutrophil infiltration is associated to blood–brain barrier breakdown and basal lamina type IV collagen degradation during hemorrhagic transformation after human ischemic stroke	379	Rosell A	Stroke	2008

### 3.3. Analysis of journals and research areas

Hot core journals can help researchers quickly find suitable journals for their articles, and also provide reliable references for researchers to retrieve literature. Analysis of the retrieved publications showed that a total of 472 journals published 1,441 manuscripts in this research area, and the top 10 journals with the most publications are shown in Table 3. First of all, the analysis of the journals from which the publications originated helps us to identify the core journals in the field. The most prolific journal is STROKE with 84 publications and an impact factor of 10.17, which shows that STROKE is a very influential journal in this field. In contrast, BRAIN RES, PLOS ONE, and NEUROSCIENCE have more publications than NEUROBIOL DIS and J NEUROINFLAMM, but the former is less influential than the latter when combined with the impact factor.

**Table 3:** The top 10 journals of publications and co-cited journals.

Rank	Journals	Publications	Impact factor (2022)	Co-cited journals	Citations	Impact factor (2022)
1	Stroke	73	10.17	Stroke	7,573	10.17
2	J Cerebr Blood F Met	66	6.96	J Cerebr Blood F Met	4,610	6.96
3	Brain Res	55	3.61	J Neurosci	2,674	6.709
4	PLoS One	31	3.752	Brain Res	2092	3.61
5	Neuroscience	27	3.708	J Biol Chem	1702	5.486
6	Neurobiol Dis	23	7.046	Circulation	1,367	39.918
7	Mol Neurobiol	22	5.682	J Neurochem	1,237	5.546
8	J Neuroinflamm	22	9.587	P Natl Acad Sci USA	1,070	12.779
9	J Neurochem	21	5.546	Nat Med	991	87.241
10	Neurol Res	21	2.529	Neuroscience	950	3.708

**Figure 5:**
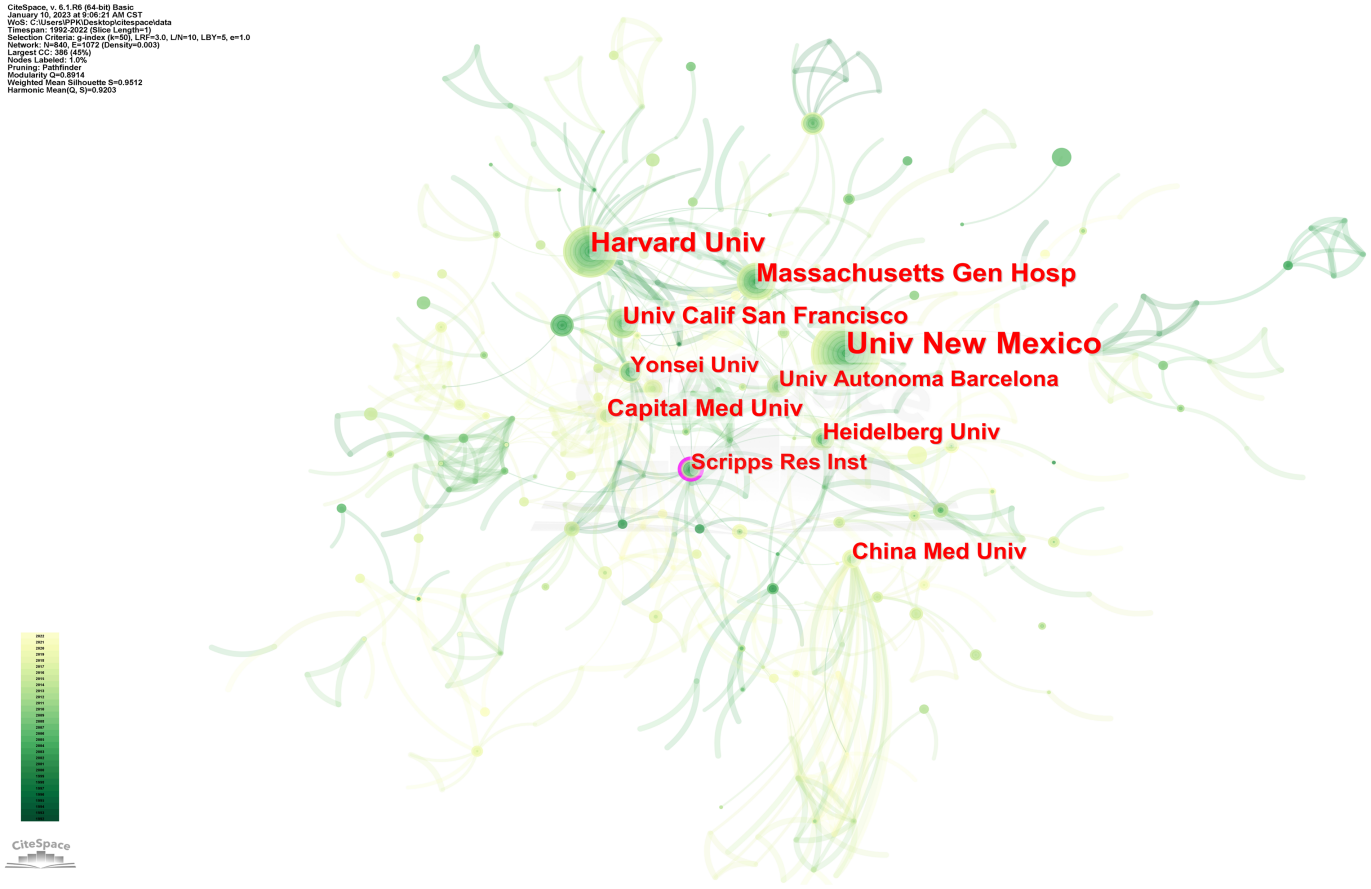
Visualization map of institutions’ cooperation network in the involvement of MMPs in IS from 1992 to 2022.

Co-cited journals are defined as the common journals that are the source of references in researchers’ articles, and they are the primary reference journals for researchers studying the involvement of MMPs in IS, reflecting side-by-side the authority and popularity of the journals among the researcher community. The names of journals of co-citation analysis were performed using VOSviewer, and the journal with a minimum number of citations over 10 was defined. As plotted in Figure 5A, 839 journals were shown in the total link strength. The top 5 journals with the best total link strength were as follows: STROKE (total link strength = 531,163 times), J CEREBR BLOOD F MET (total link strength = 369,287 times), J NEUROSCI (total link strength = 223,352 times), BRAIN RES (total link strength = 167,397 times), and J BIOL CHEM (total link strength = 164,345 times).

Using CiteSpace for the dual-map overlay of journals (Figure 5B). The left side of the figure shows the cited journals and the right side shows the reference journals. The spline waves from left to right depict the citation association, which is represented by the colored paths. As can be seen from the figure, the main areas of research include clinical, medical, neurology, immunology and biochemistry molecular biology, etc. In addition, a major citation pathway marked in orange is depicted in the figure, which indicates that journals in molecular/biology/genetics (e.g., J Neurosci, J Neurochem, and P Natl Acad Sci USA, etc.) are mainly cited by journals in molecular/biology/immunology (e.g., J Cerebr Blood F Met and Neuroscience, etc.).

### 3.4. Analysis of authors and co-authorship

From January 1, 1992, to December 31, 2022, a total of 6,646 authors were involved in the study of the role of MMPs in IS. The top 10 authors with the highest number of publications are listed in Table 4. The evaluation criteria for core authors in this field include the number of publications in the field, the number of citations in the field, and the H-index. Accordingly, Rosenberg GA, Lo EH, and Montaner J are known to be core authors in the field.

**Table 4:** The top 10 authors.

Rank	Authors	Countries	Affiliations	Publications	Citations	H-index
1	Rosenberg GA	United States	University of New Mexico	49	2,144	41
2	Lo EH	United States	Massachusetts General Hospital	41	1781	34
3	Montaner J	Spain	Vall d’Hebron Hospital	33	866	24
4	Rosell A	Spain	Vall d’Hebron Hospital	21	538	17
5	Yang Y	United States	University of New Mexico	21	599	20
6	Wang XY	United States	Massachusetts General Hospital	18	750	16
7	Lee SR	United States	Massachusetts General Hospital	17	583	17
8	Del Zoppo GJ	United States	The Scripps Research Institute	15	709	13
9	Candelario-Jalil E	United States	University of New Mexico	15	274	15
10	Wang J	United States	The Johns Hopkins University	15	126	13

Co-authorship analysis was performed using Bibliometrix for high-producing authors with ≥6 publications, and the results are shown in Figure 3. As can be seen from the figure and table, there is frequent collaboration and close contact between Rosenberg GA, Lo EH, and Montaner J on related research, and they are leaders and widely influential in the field.

### 3.5. Analysis of institution and institutional cooperation

The analysis showed that these publications came from a total of 1,492 institutions, and the top 10 institutions with the most publications are shown in Table 5. Among these institutions, University of New Mexico, Harvard University, Massachusetts General Hospital, University of California, San Francisco, and Scripps Research Institute are all from the United States, indicating that the relevant institutions in the United States have strong research strengths in studying the role of MMPs in IS. They are in the leading position in this field. In terms of start year, institutions from the United States started the earliest (1998–2002), while Capital Medical University from China started the latest (2009). However, it is worth noting that institutions from China, such as CAPITAL MED UNIV and CHINA MED UNIV, are developing rapidly in studying the role of MMPs in IS and may become the backbone of the field in the future.

**Table 5:** The top 10 institutions.

Rank	Institutions	Publications	Starting year
1	University of New Mexico	65	1998
2	Harvard University	44	2000
3	Massachusetts General Hospital	39	2000
4	Universitat Autònoma de Barcelona	27	2005
5	Yonsei University	25	2003
6	University of California, San Francisco	24	2003
7	Capital Medical University	22	2008
8	Heidelberg University	21	1999
9	China Medical University	20	2003
10	Scripps Research Institute	20	1999

To present the cooperation among institutions, CiteSpace was used to generate a network graph of institutional cooperation, with the number of years per slice set to 1, the results are shown in Figure 5. The larger the circle, the greater the number of publications. The lines between the circles indicate cooperative relationships, and the thickness indicates the strength of the links between institutions. Network density is the ratio of the actual number of lines in the network to the theoretical maximum number of lines. Intermediary centrality is a measure of the importance of nodes in a network, and CiteSpace uses this metric to discover and measure the importance of nodes and uses pink circles to highlight such nodes. The thicker the outermost circle, the greater the centrality, which implies frequent collaboration between institutions. According to the figure, the collaboration between different institutions in this field is generally close with a network density of 0.003. In particular, there is a strong collaboration between Harvard University and Massachusetts General Hospital.

### 3.6. Analysis of countries and international cooperation

From January 1, 1992, to December 31, 2022, 60 countries and regions are involved in this area of research. The country node in CiteSpace software was selected to generate a country or regional cooperation network map with the number of years per slice set to 1. The results are shown in Figure 5A. The larger the circle, the greater the number of publications. The lines between the circles indicate cooperative relationships, and the thickness indicates the strength of the links between countries and regions. The thicker the pink circle, the greater the centrality, which implies frequent cooperation between countries and regions. According to the figure, the cooperation between countries or regions in this field is generally close, with a network density of 0.1237. The country collaboration map shows that cooperation exists mainly in North America, Western Europe, and East Asia. In general, many countries or regions tend to cooperate and communicate with each other. The research area of the role of MMPs in IS has a strong global network character.

**Figure 6:**
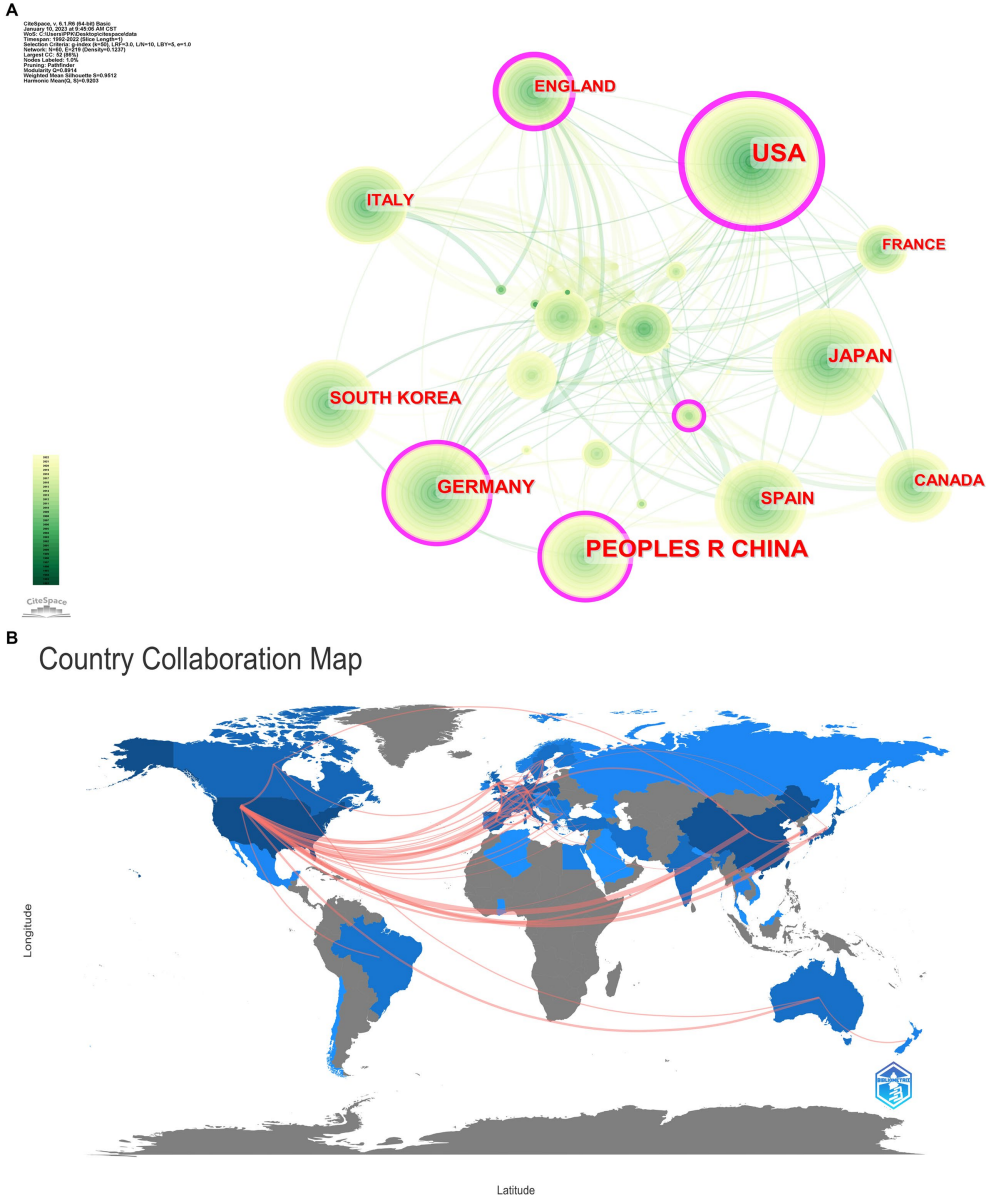
**(A)** Visualization map of countries/regions cooperation network in the involvement of MMPs in IS from 1992 to 2022. **(B)** Country collaboration map.

The top 10 most productive countries and regions are listed in Table 6. The countries or regions with more than 100 publications include the United States, China, Japan, and Germany, among which the number of publications of United States and China significantly exceeds other countries, indicating that the United States and China have fruitful research results and strong research strength in this field. In addition, the United States ranked first with a centrality of 0.53, indicating that the United States plays the most important role in the knowledge transfer process of the role of MMPs in IS. In addition to the United States, countries with centrality >0.1 include England, Germany, and China, which also play an important role in the process of knowledge transfer in this field. In terms of start year, the United States was the earliest, in 1992, while China and South Korea started later, in 2003. However, it is worth noting that China and South Korea are quite active in this field, and they may become stalwarts in this field in the future (Figure 6).

**Table 6:** Top 10 countries/regions according to publications.

Rank	Country	Publications	Centrality	Starting year
1	United States	585	0.53	1992
2	China	348	0.13	2003
3	Japan	109	0.04	2000
4	Germany	107	0.16	1999
5	South Korea	84	0.01	2003
6	Spain	75	0.09	2000
7	Italy	61	0.06	2001
8	England	50	0.21	2001
9	Canada	49	0.07	1997
10	France	43	0.02	2001

### 3.7. Analysis of keywords

**Figure 7:**
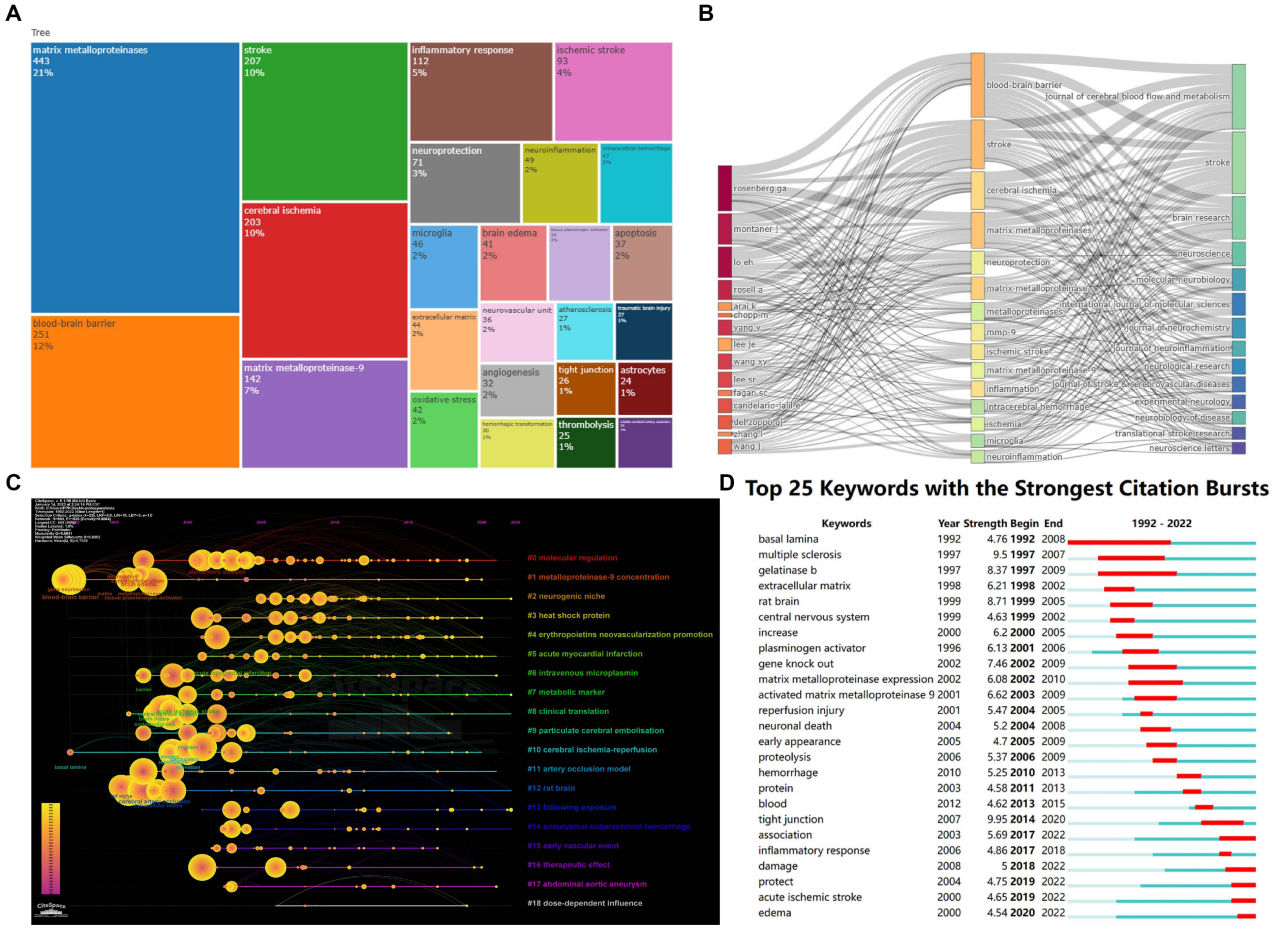
**(A)** Treemap showing the top 25 most frequently occurring keywords. **(B)** In the three-field plot, the left field is the authors, the middle field is the keywords, the right field is the journals, and the width of the connecting lines represents their correlation strength. **(C)** Keyword clustering timeline Graph made by CiteSpace. **(D)** Burst keywords captured by CiteSpace bursts detection.

Keywords demonstrate the thematic concepts and core ideas of a paper and can briefly describe specific research hotspots. The 25 most frequently occurring keywords are shown in Figure 7A. In addition, analyzing the connections between keywords and authors, and The connections between keywords and journals can help us select the most popular keywords. Figure 7B represents a three-field plot, through which the linkage between keywords, authors, and journals can be observed, and the width of the connecting lines represents their correlation strength. It can be seen that the “blood–brain barrier,” “stroke” and “cerebral ischemia,” the three keywords are closely associated with the authors Rosenberg GA, Montaner J, Lo EH, and Rosell A, which can indicate both the research focus of these authors in the field and the popularity of these keywords by authors and journals.

A timeline graph is constructed using CiteSpace to visualize keyword clustering (Figure 7C). Each circle in the graph represents a keyword, each line is a timeline, the circles near the line are the same class, and the length of the line represents the time span of the keyword appearing in the same class. It can be seen that the keywords are divided into 19 categories, among which, “molecular regulation” (cluster 0), “metalloproteinase-9 concentration” (cluster 1), “clinical translation” (cluster 8), “cerebral ischemia–reperfusion” (cluster 10) and “rat brain” (cluster 12) are the points that have received more attention since 1992.

Using CiteSpace to capture burst keywords, which are indicators of leading-edge topics over time, the intensity and duration of the top 25 keywords with the highest burst intensity are shown in Figure 7D. Based on the keyword hotspot trend graph, it can be seen that the explosive research is mainly focused on brain ischemia–reperfusion injury, neuronal cell death, central nervous system (CNS) inflammatory response, and the BBB. In addition, it can be seen that MMP-9 is widely emphasized in related studies. The analysis also shows that more researchers have shifted their attention to the role of MMPs in the acute phase after IS.

## 4. Discussion

As ubiquitous proteins, MMPs have a wide range of roles. Over the past few decades, scientists have made great efforts to understand the role of MMPs in IS, and great advances have been made in the treatment of MMPs-related mechanisms. The role of MMPs in the complex physiopathology of IS has been widely recognized. With the help of the emerging science of bibliometrics, we can gain a deeper understanding of the state of research in specific fields and predict future trends. To our knowledge, this is the first study to examine the association between MMPs and IS based on bibliometrics and visual analysis.

According to Figure 2, during the 20 years from 1992 to 2012, the number of MMPS-related publications in IS increased steadily on the whole, reaching a small peak in 2012. In the following 5 years, the number of MMPs-related publications in IS began to decrease, but it can be seen from the still high number of publications that related topics still have a certain research heat. However, in the last 5 years, the number of publications on this topic has significantly increased and may continue to decrease in the future. Combining the results of bibliometric analysis, we can infer the reasons for this trend. After the first paper on the subject was published in 1992, research on the subject did not make a splash. As can be seen from the annual growth of publications and the top 10 highly cited articles in Table 2B, it was not until 1998 that Stroke published two articles on the role of MMPs in cerebral ischemia, which attracted the attention of researchers and led to an increase in annual publications. These two influential papers suggested that the expression of matrix metalloproteinase was increased after focal cerebral ischemia in rats, and MMP-2 and MMP-9 were mainly increased. The infarct size of the animals was significantly reduced when MMP-9 was neutralized by a monoclonal antibody. It has also been suggested that MMPs are associated with the opening of the BBB after brain reperfusion in rats (Romanic et al., 1998; Rosenberg et al., 1998). These laid the foundation for the following research. Moreover, in 2002, Science published the first article in the top ten cited, further proving the mechanism of MMPs in cerebral ischemia, MMP-9 can be activated by S-nitrosylation during cerebral ischemia, thus inducing neuron apoptosis (Gu et al., 2002). Five years later, based on the above studies, some scholars believe that, contrary to the damage in the acute phase of stroke, MMP-9 can promote neurovascular remodeling through VEGF 7 days after stroke (Zhao et al., 2006). Since MMPs are associated with intravenous recombinant tissue plasminogen activator-mediated delayed reperfusion mortality and early opening of BBB (Pfefferkorn and Rosenberg, 2003), the mechanism of MMPs in the opening of BBB in cerebral ischemia–reperfusion injury was further investigated. Activation of MMP-2 is involved in the early destruction of tight junction proteins (TJP), leading to the initial reversible opening of BBB (Zhang et al., 2018). However, with the passage of time and increased neuroinflammation, MMP-9 plays an important role in the delayed damage of BBB. The ability of broad-spectrum MMP inhibitors to reduce BBB damage by reducing the degradation of TJP (Yang et al., 2007). Although the number of citations is somewhat related to the length of publication, the top 10 articles are somewhat related to the annual growth of publications on related topics from the time node of publication. The interval between milestone years suggests that it may take about 5 years to achieve a breakthrough in this area. Therefore, there is an urgent need for new research directions to continue to drive the research of MMPs in IS.

A visual analysis of national/regional contributions shows that the United States has a clear advantage in MMPs and IS research. The United States accounts for more than half of all publications on the subject. The United States has a maximum centrality of 0.53, indicating that they play a central role in international collaboration on research on this topic. In the analysis of institutions, it is also found that American institutions account for more than half of the top 10 institutions in the number of publications produced. The United States was also the first country to start this research, which shows that the United States is highly productive and a leader in this field. Although China started research in this field much later than the United States, the total number of publications has exceeded most other countries, ranking second after the United States. Research institutions from China, which have developed rapidly in studying the role of MMPs in IS, may become the backbone of the field in the future. However, it still needs many years of effort to surpass the United States, a powerful scientific research country.

Next, we identified influential experts in the field of MMPs in IS through author and co-author analysis. Rosenberg GA has 49 articles on this research topic, with a total of 2,144 citations and an H-index of 41, making him the core author in this field. The two publications with the most local citations under the topic are also his articles. He started looking at MMPs in brain injury in 1995, not long after the field had been studied (Rosenberg, 1995). Since then, he has focused on the role of MMPs in neurological diseases and, more specifically, in neuronal death or BBB integrity in ischemic disease. This author is recognized by the largest number of researchers in the field for making outstanding contributions.

More information can be obtained based on keyword analysis and further literature reading. The BBB is the earliest, longest, and highest proportion of MMP research keywords in the topic of cerebral ischemia–reperfusion. BBB integrity plays a crucial role in maintaining the normal physiology of the brain. Intense neuroinflammation in the acute phase of IS is associated with BBB breakdown and worse neurologic outcomes, and the mechanism of injury driven by inflammation in IS includes increased production of MMPs (Rosenberg et al., 1998; Zhang et al., 2018; Yang et al., 2019). MMP-2(gelatinase A) and MMP-9(gelatinase B) have been the focus of BBB studies in cerebral ischemia due to their ability to degrade the basement membrane due to their substrate specificity for the major components of the perivascular basement membrane, collagen IV, laminin, and fibronectin. But in mice, MMP-9 plays a dominant role, because MMP-9 knockout mice are protected from ischemia stroke, whereas MMP-2 knockout mice were not protected (Wang et al., 2000; Asahi et al., 2001). In the hemorrhagic transformation complication of tPA, the only thrombolytic drug for IS, MMP-9 is also related to the destruction of BBB. rtPA significantly induces MMP-9 release from human neutrophils and contributes to hemorrhagic transformation after thrombolytic therapy in IS (Cuadrado et al., 2008).

MMP-9, as separate MMPs in the first few keywords, is confirmed that it is the most widely studied MMP. The level of MMP-9 is significantly increased after stroke onset, and the level is correlated with infarct size, stroke severity, and functional outcome. MMP-9 is a possible marker of persistent cerebral ischemia (Ramos-Fernandez et al., 2011). In addition to the role of brain ischemia injury BBB damage research, it’s also associated with excitatory toxicity and neuronal damage. Pharmacological or genetic knockout approaches have shown that MMP-9 contributes to the delay of hippocampal neuronal death after transient global cerebral ischemia (Lee et al., 2004). Elevated MMP-2 and MMP-9 activity degrades nuclear DNA repair proteins and promotes the accumulation of oxidative DNA damage in neurons, thus promoting neuronal death after OGD/R (Yang et al., 2010). A large number of studies have also focused on finding ways to inhibit MMP-9 activity to reduce cerebral ischemia–reperfusion injury (Chaturvedi and Kaczmarek, 2014). However, due to the lack of low-toxicity selective inhibitors, targeting MMP-9 to reduce stroke damage has been proven to be difficult (Candelario-Jalil et al., 2022). Fortunately, a potential feasible therapy has recently been discovered. Human IgG monoclonal antibody (mAb) L13 has specificity for neutralizing MMP-9 and has therapeutic effects in mouse stroke models. It can also significantly neutralize the enzymatic activity of MMP-9 in stroke patient samples (Ji et al., 2023). Furthermore, due to the excessive attention paid to BBB and MMP-9 in previous studies, exploring the mechanism of other MMPs in cerebral ischemia–reperfusion besides BBB destruction may be an idea for future research.

There are still some limitations to be discussed. First, the data in this study only included articles and reviews in English from SCI-Expanded. Second, we excluded papers published in 2023, so we might neglect some influential newly published studies. Therefore, a bibliometric analysis of newly published articles should be continued at specific times to enrich the findings in this area continuously. Third, due to the limitation of bibliometric software, the results obtained by using different software for data statistics may be biased. Therefore, more powerful software is recommended in future research.

## 5. Conclusion

This study provides knowledge about IS and MMPs from a bibliometric perspective. The results suggest that research on the role of MMPs in IS has become increasingly widespread worldwide in the last 30 years. The rapid growth trend of publications on this topic from 1998 to 2012 indicates the growing interest of scholars during this period, but at present, given the gradual receding of the topic in the last 5 years, its enthusiasm is likely to maintain a downward trend in the future as well. In addition, keyword clustering analysis showed that among the MMPs, the most popular was MMP-9, and scholars delved into the direction of ischemia–reperfusion injury, neuronal cell death, neuroinflammation, BBB, and the role of MMPs in acute IS. Overall, the study of the role of MMPs in IS has made great progress in the last 30 years, and the present study can provide valuable insights to researchers through bibliometric analysis and enable them to obtain meaningful references based on objective data.

## Data availability statement

The original contributions presented in the study are included in the article/Supplementary material, further inquiries can be directed to the corresponding author.

## Author contributions

YG performed data retrieval, analysis and visualization. YL completed the writing of the article. SF helped to prepare the manuscript. LG designed the manuscript and prepared the final version. All authors contributed to the article and approved the submitted version.

## Conflict of interest

The authors declare that the research was conducted in the absence of any commercial or financial relationships that could be construed as a potential conflict of interest.

## Publisher’s note

All claims expressed in this article are solely those of the authors and do not necessarily represent those of their affiliated organizations, or those of the publisher, the editors and the reviewers. Any product that may be evaluated in this article, or claim that may be made by its manufacturer, is not guaranteed or endorsed by the publisher.
